# Supplementary material for: Formation of Aggregate-Free Gold Nanoparticles in the Cyclodextrin-Tetrachloroaurate System Follows Finke–Watzky Kinetics
Source: Nanomaterials (Basel). 2022 Feb 9;12(4):583. doi: 10.3390/nano12040583 (PMC8875903; doi:10.3390/nano12040583)
Supplement: Supplementary file 1 [file nanomaterials-12-00583-s001.zip › nanomaterials-1572588-supplementary.pdf]

Supplementary Materials

Formation of Aggregate-Free Gold Nanoparticles in the Cyclodextrin-Tetrachloroaurate System Follows Finke–Watzky Kinetics

Yuri Sergeyevich Pestovsky [1,2,*] and Teerapol Srichana [1,3,*]

[1] Drug Delivery System Excellence Center, Prince of Songkla University, Songkhla 90110, Thailand
[2] Genetic Engineering Department, Center for Research and Advanced Studies of the National Polytechnic Institute (Cinvestav), Irapuato 36824, Mexico
[3] Faculty of Pharmaceutical Sciences, Prince of Songkla University, Songkhla 90110, Thailand
* Correspondence: upestovskij@gmail.com (Y.S.P.); teerapol.s@psu.ac.th (T.S.); Tel.: +66-7428-8979 (T.S.)

Table S1. Recommended conditions for the synthesis of aggregate-free gold nanoparticles with immobilized cyclodextrins at room temperature.

Cyclodextrin		pH	Time,	Centrifugation parameters		Average particle size,
Type	Concentration, M		min	Speed, g	Duration, min	nm
α	0.004	10.56	149 – 184; 768	7430	20	12-13
	0.002	11.5	413 – 613	7430	20	23-29
	0.004	11.5	288; 435 – 966	7430	20	1; 18; 22-25
β	0.004	10.56	93 – 210; 1172	7430	20	11.5-13
		11	195 – 546	13200	10	14-16
		11.5	125 – 280	7430	20	1; 37-42

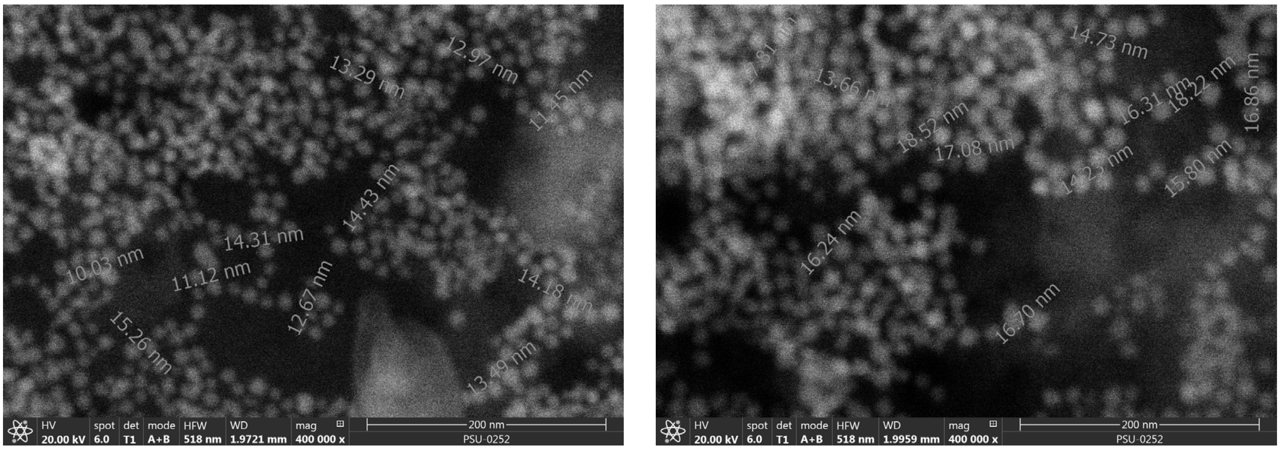

(**a**) (**b**)

Figure S1. Scanning electron microscopy images of gold nanoparticles with immobilized **(a)** α-cyclodextrin and **(b)** γ-cyclodextrin synthesized under reflux.

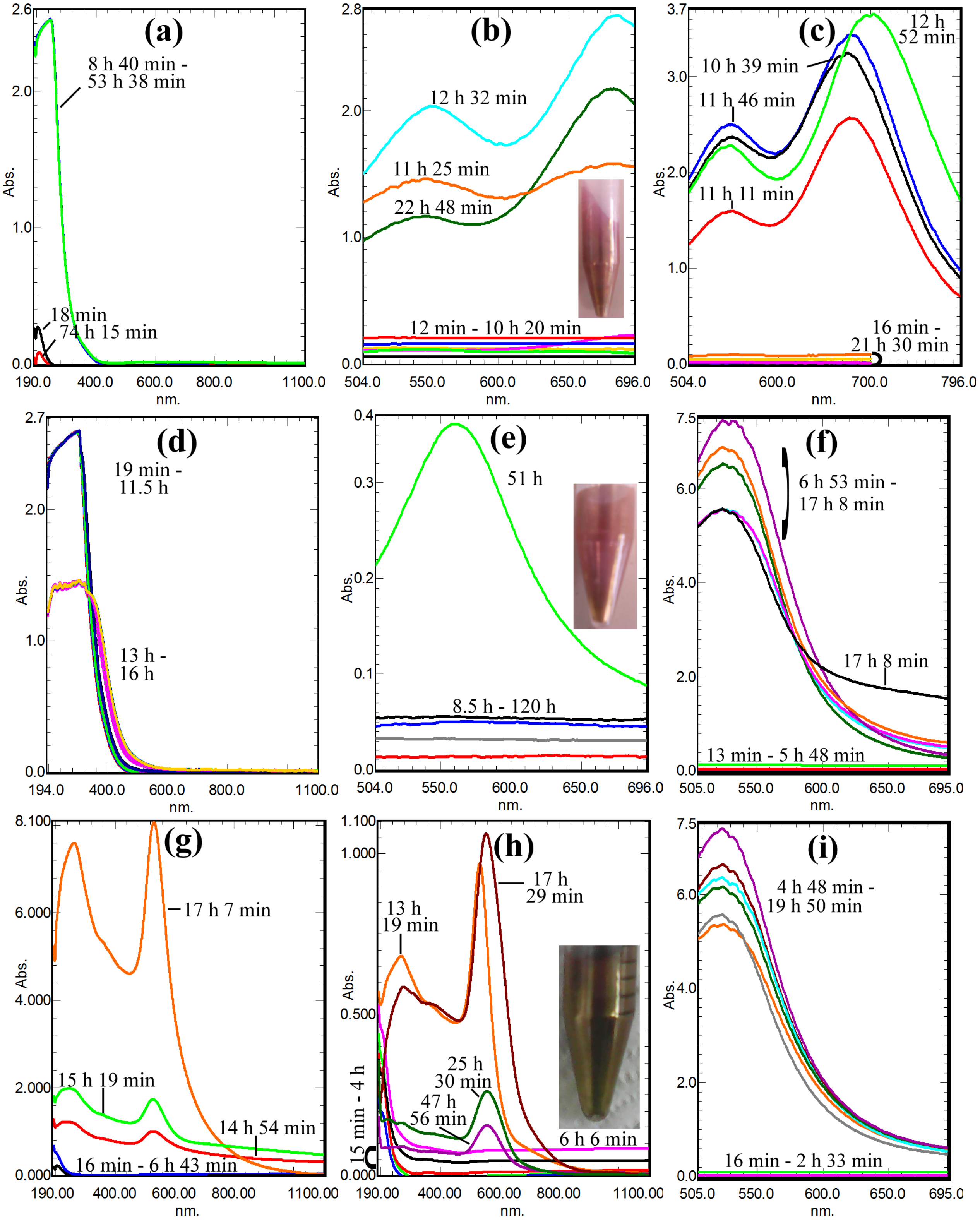
(a)
8 h 40 min -
53 h 38 min
18 min
74 h 15 min
Abs.
nm.
(b)
12 h 32 min
11 h 25 min
22 h 48 min
12 min - 10 h 20 min
(c)
12 h
52 min
10 h 39 min
11 h 46 min
11 h 11 min
16 min -
21 h 30 min
(d)
19 min -
11.5 h
13 h -
16 h
(e)
51 h
8.5 h - 120 h
(f)
6 h 53 min -
17 h 8 min
17 h 8 min
13 min - 5 h 48 min
(g)
17 h 7 min
15 h 19 min
14 h 54 min
16 min - 6 h 43 min
(h)
17 h
29 min
13 h
19 min
25 h
30 min
47 h
56 min
15 min - 4 h
6 h 6 min
(i)
4 h 48 min -
19 h 50 min
16 min - 2 h 33 min

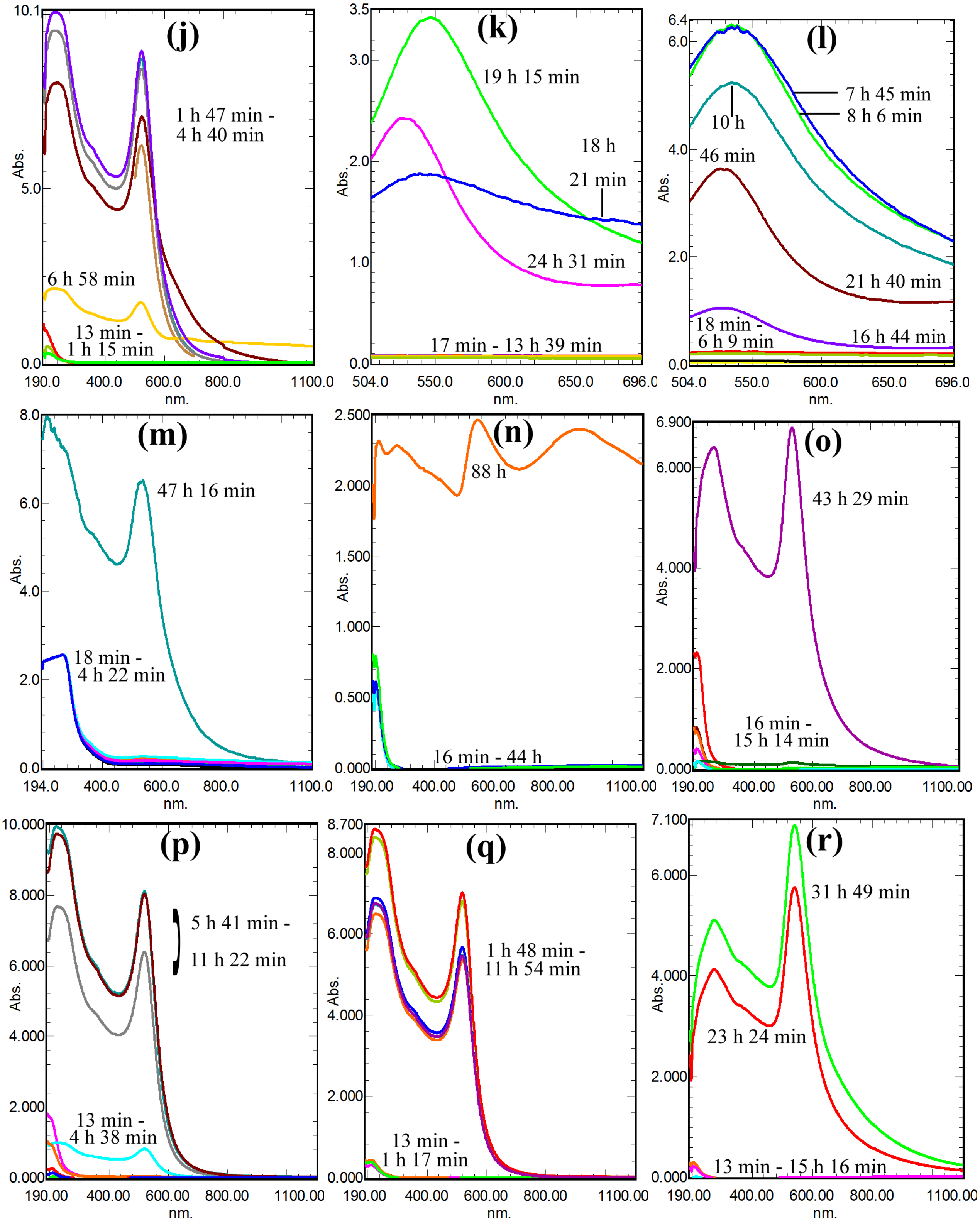
(j)
1 h 47 min -
4 h 40 min
6 h 58 min
13 min -
1 h 15 min
Abs.
nm.
(k)
19 h 15 min
18 h
21 min
24 h 31 min
17 min - 13 h 39 min
Abs.
nm.
(l)
7 h 45 min
8 h 6 min
10 h
46 min
21 h 40 min
18 min -
6 h 9 min
16 h 44 min
Abs.
nm.
(m)
47 h 16 min
18 min -
4 h 22 min
Abs.
nm.
(n)
88 h
16 min - 44 h
Abs.
nm.
(o)
43 h 29 min
16 min -
15 h 14 min
Abs.
nm.
(p)
5 h 41 min -
11 h 22 min
13 min -
4 h 38 min
Abs.
nm.
(q)
1 h 48 min -
11 h 54 min
13 min -
1 h 17 min
Abs.
nm.
(r)
31 h 49 min
23 h 24 min
13 min - 15 h 16 min
Abs.
nm.

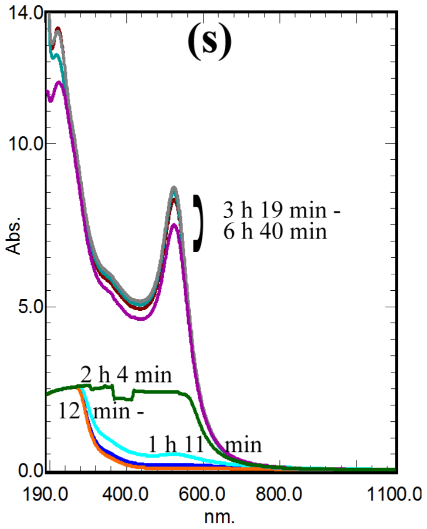

Figure S2. Absorbance spectra of gold nanoparticles synthesized at 0.1% (w/w) $HAuCl_4$ at room temperature **(a)** at 0.001 M γ-cyclodextrin, pH 10.56; at pH 12: **(b)** at 0.004 M α-cyclodextrin; **(c)** at 0.004 M β-cyclodextrin; **(d)** at 0.004 M γ-cyclodextrin; at pH 11.5: **(e)** at 0.001 M α-cyclodextrin; **(f)** at 0.002 M α-cyclodextrin; **(g)** at 0.002 M α-cyclodextrin in the absence of HCl; **(h)** at 0.002 M α-cyclodextrin in the presence of NaCl; **(i)** at 0.004 M α-cyclodextrin; **(j)** at 0.004 M β-cyclodextrin; **(k)** at 0.001 M γ-cyclodextrin; **(l)** at 0.002 M γ-cyclodextrin; **(m)** at 0.004 M γ-cyclodextrin; at pH 11: **(n)** at 0.001 M α-cyclodextrin; **(o)** at 0.002 M α-cyclodextrin; **(p)** at 0.004 M α-cyclodextrin; **(q)** at 0.004 M β-cyclodextrin; **(r)** at 0.002 M γ-cyclodextrin; **(s)** at 0.004 M γ-cyclodextrin. The respective 'golden mirrors' are shown as insets.

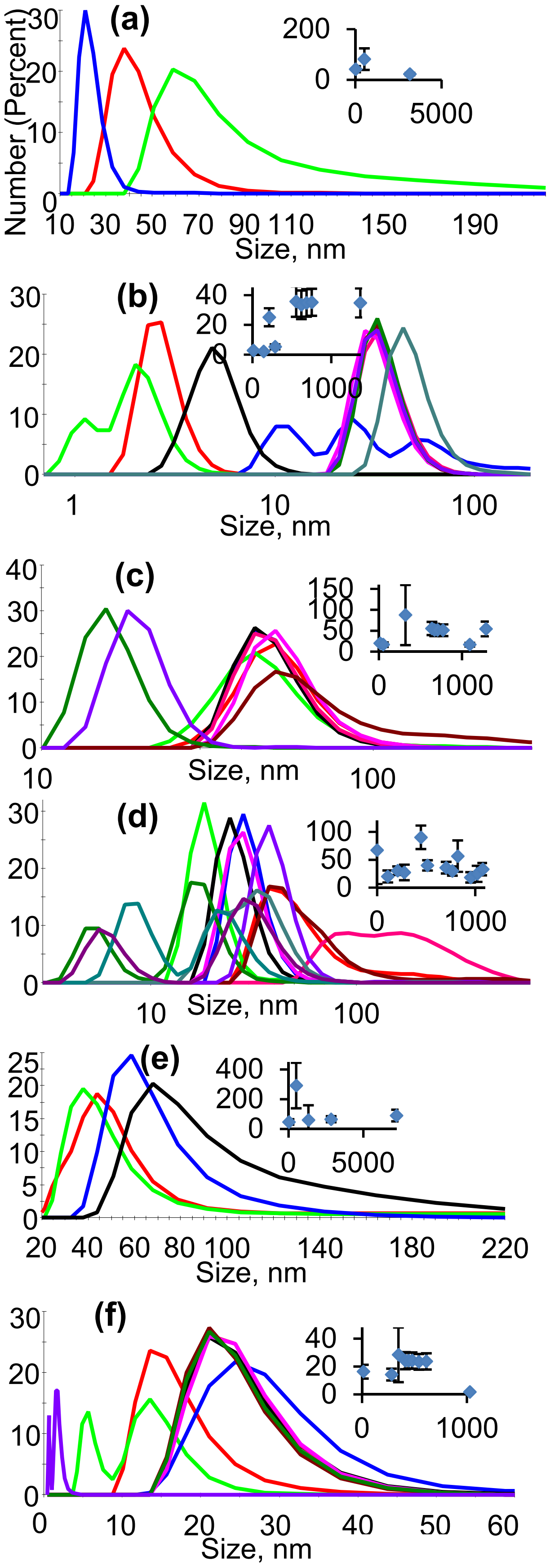
(a)
(b)
(c)
(d)
(e)
(f)
Number (Percent)
Size, nm
Size, nm
Size, nm
Size, nm
Size, nm
Size, nm

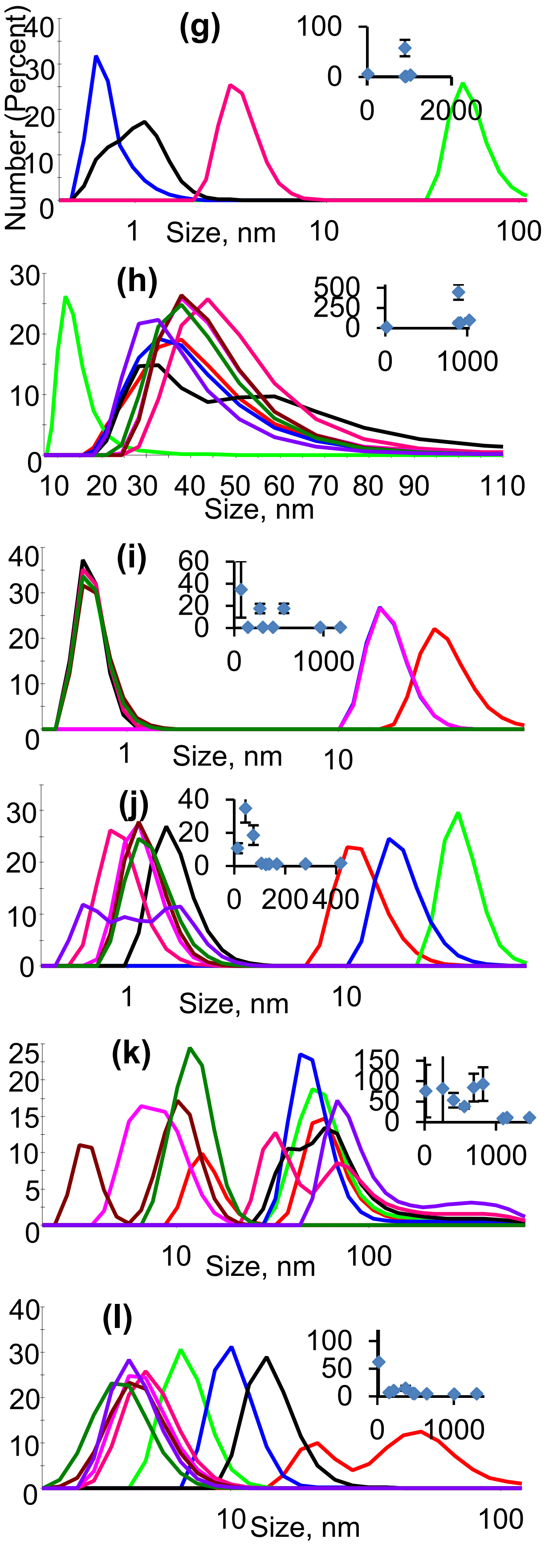
(g)
(h)
(i)
(j)
(k)
(l)
Number (Percent)
Size, nm

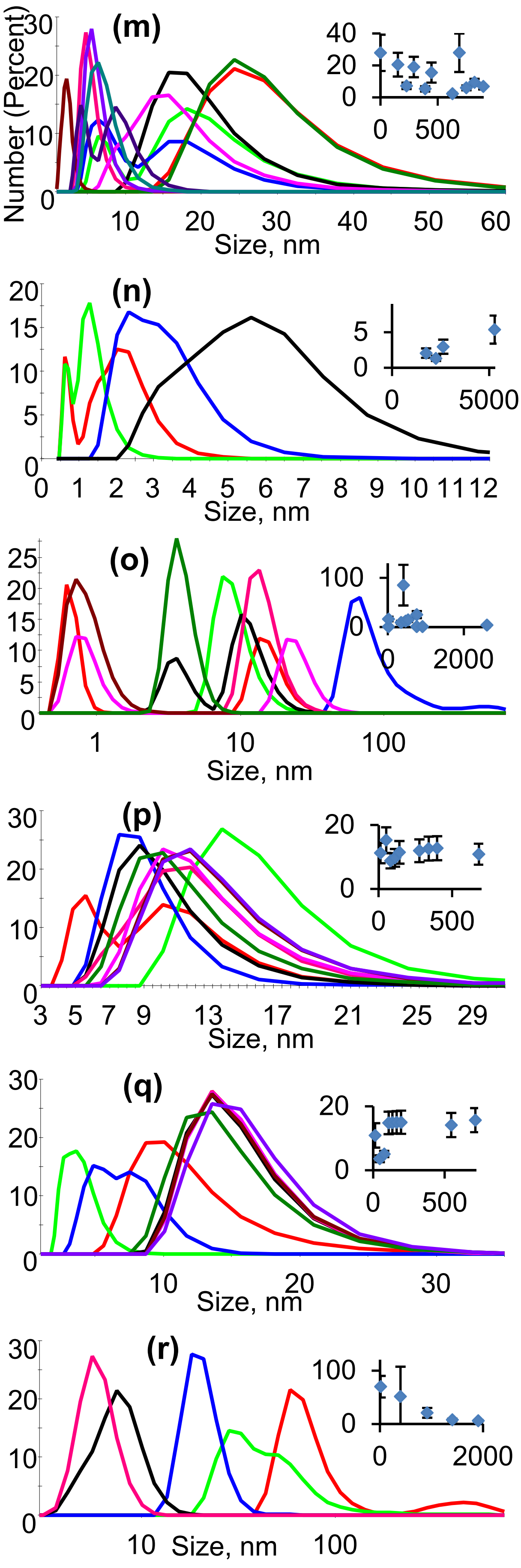
(m)
Number (Percent)
Size, nm
(n)
Size, nm
(o)
Size, nm
(p)
Size, nm
(q)
Size, nm
(r)
Size, nm

Figure S3. Number size distributions of gold nanoparticles synthesized at 0.1% (w/w) $HAuCl_4$ at room temperature **(a)** at 0.001 M γ-cyclodextrin, pH 10.56; at pH 12: **(b)** at 0.004 M α-cyclodextrin; **(c)** at 0.004 M β-cyclodextrin; **(d)** at 0.004 M γ-cyclodextrin; at pH 11.5: **(e)** at 0.001 M α-cyclodextrin; **(f)** at 0.002 M α-cyclodextrin; **(g)** at 0.002 M α-cyclodextrin in the absence of HCl; **(h)** at 0.002 M α-cyclodextrin in the presence of NaCl; **(i)** at 0.004 M α-cyclodextrin; **(j)** at 0.004 M β-cyclodextrin; **(k)** at 0.001 M γ-cyclodextrin; **(l)** at 0.002 M γ-cyclodextrin; **(m)** at 0.004 M γ-cyclodextrin; at pH 11: **(n)** at 0.001 M α-cyclodextrin; **(o)** at 0.002 M α-cyclodextrin; **(p)** at 0.004 M α-cyclodextrin; **(q)** at 0.004 M β-cyclodextrin; **(r)** at 0.002 M γ-cyclodextrin. The data were acquired in back-scattering mode. The insets show the dependence of the main number size distribution peak (nm) on reaction time (min). Samples containing only aggregates were omitted from the main plots but not from the insets.

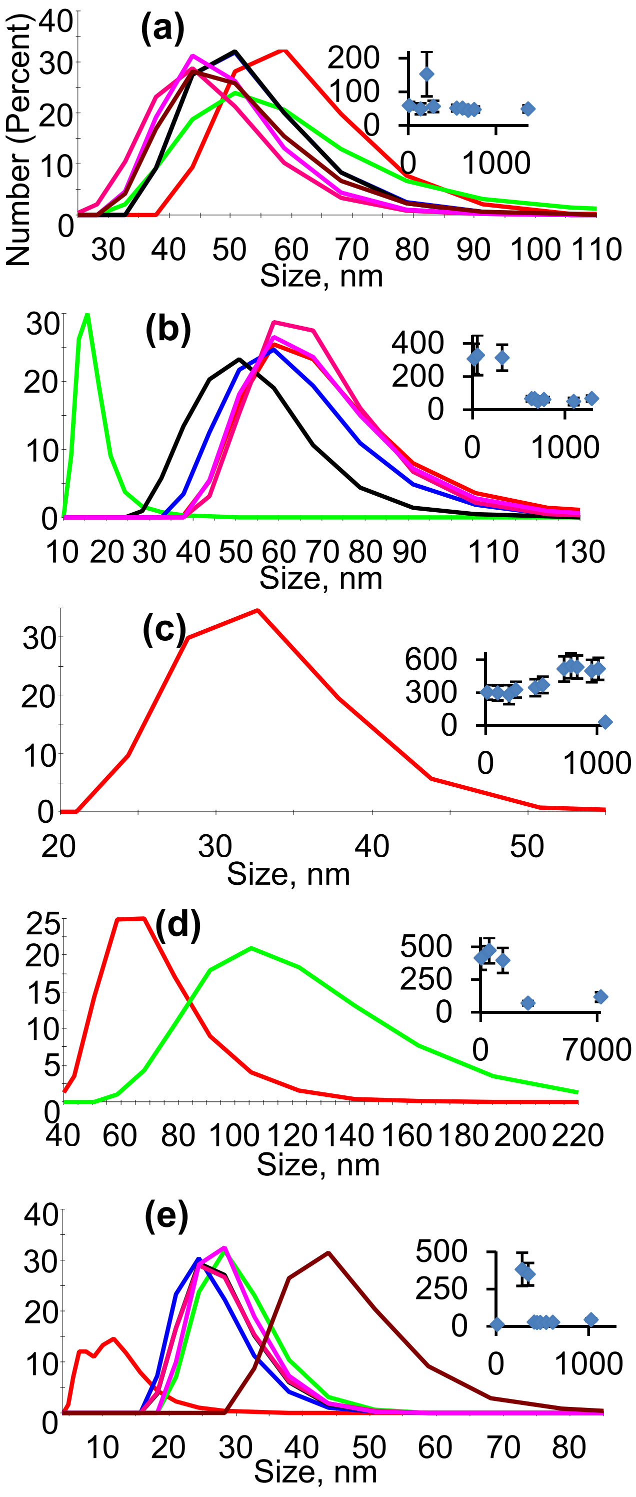

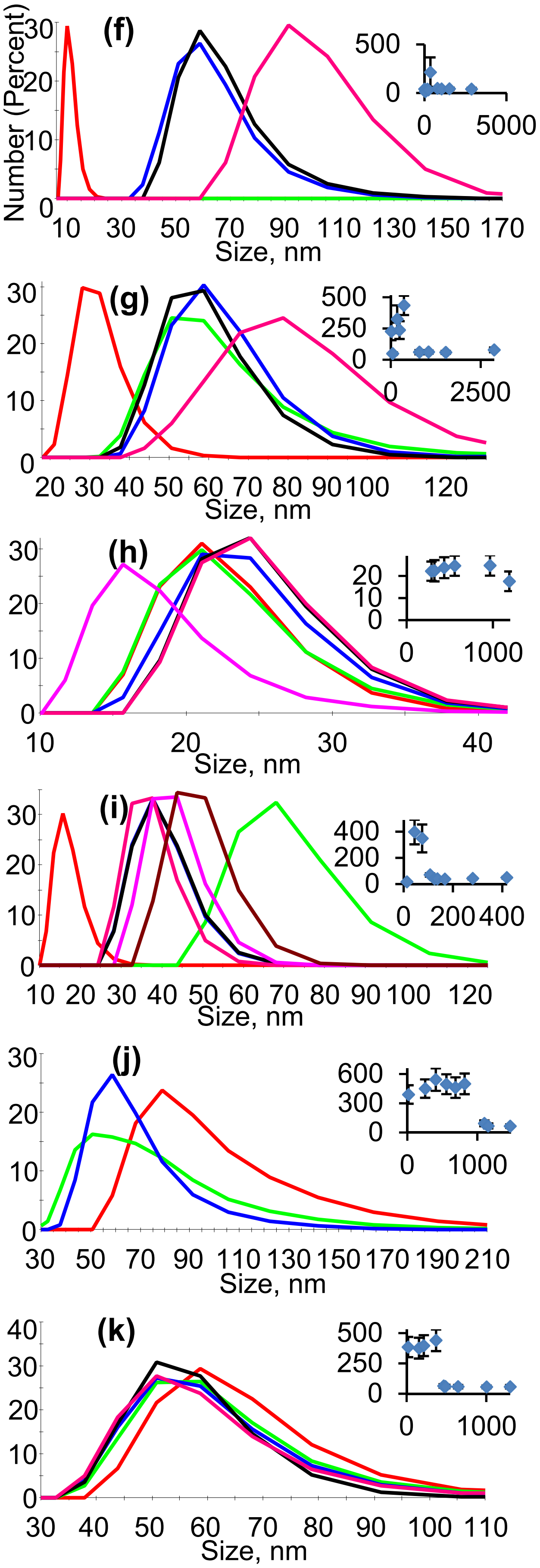
(f)
Number (Percent)
0
10
20
30
10 30 50 70 90 110 130 150 170
Size, nm
500
0
0 5000
(g)
0
10
20
30
20 30 40 50 60 70 80 90 100 120
Size, nm
500
250
0
0 2500
(h)
0
10
20
30
10 20 30 40
Size, nm
20
10
0
0 1000
(i)
0
10
20
30
10 20 30 40 50 60 70 80 90 100 120
Size, nm
400
200
0
0 200 400
(j)
0
10
20
30
30 50 70 90 110 130 150 170 190 210
Size, nm
600
300
0
0 1000
(k)
0
10
20
30
40
30 40 50 60 70 80 90 100 110
Size, nm
500
250
0
0 1000

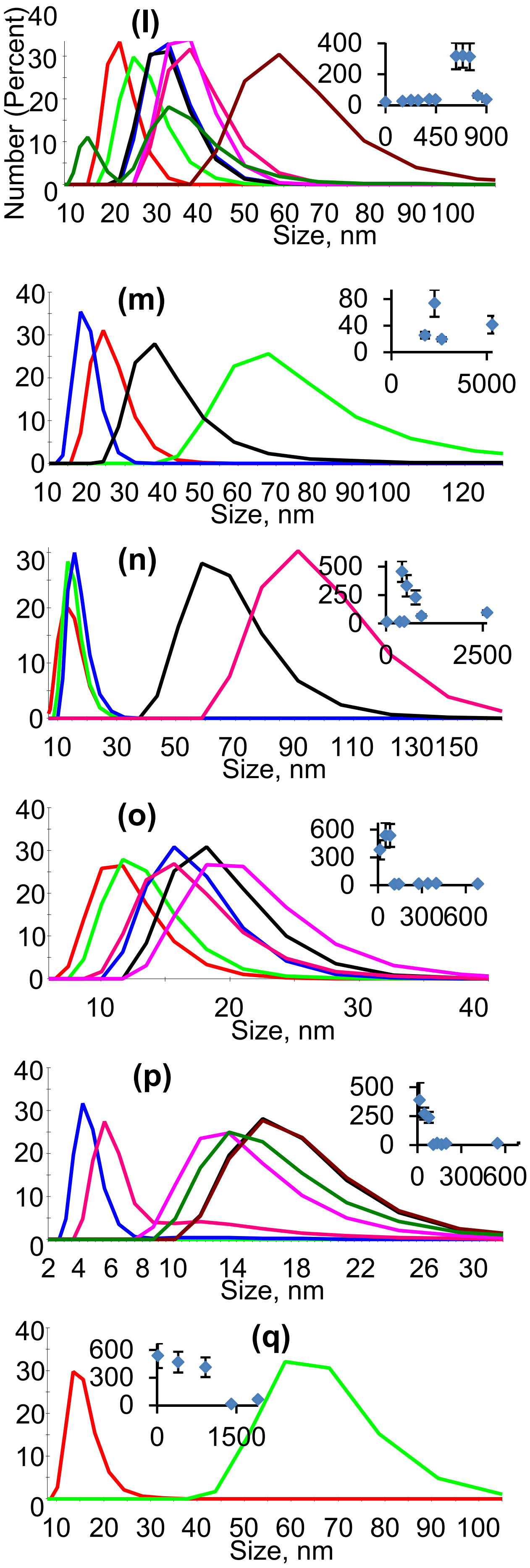
(l)
Number (Percent)
0 10 20 30 40
10 20 30 40 50 60 70 80 90 100
Size, nm
400 200 0
0 450 900
(m)
0 10 20 30 40
10 20 30 40 50 60 70 80 90 100 120
Size, nm
80 40 0
0 5000
(n)
0 10 20 30
10 30 50 70 90 110 130 150
Size, nm
500 250 0
0 2500
(o)
0 10 20 30 40
10 20 30 40
Size, nm
600 300 0
0 300 600
(p)
0 10 20 30 40
2 4 6 8 10 14 18 22 26 30
Size, nm
500 250 0
0 300 600
(q)
0 10 20 30 40
10 20 30 40 50 60 70 80 90 100
Size, nm
600 300 0
0 1500

Figure S4. Number size distributions of gold nanoparticles synthesized at 0.1% (w/w) $HAuCl_4$ at room temperature at pH 12: **(a)** at 0.004 M α-cyclodextrin; **(b)** at 0.004 M β-cyclodextrin; **(c)** at 0.004 M γ-cyclodextrin; at pH 11.5: **(d)** at 0.001 M α-cyclodextrin; **(e)** at 0.002 M α-cyclodextrin; **(f)** at 0.002 M α-cyclodextrin in the absence of HCl; **(g)** at 0.002 M α-cyclodextrin in the presence of NaCl; **(h)** at 0.004 M α-cyclodextrin; **(i)** at 0.004 M β-cyclodextrin; **(j)** at 0.001 M γ-cyclodextrin; **(k)** at 0.002 M γ-cyclodextrin; **(l)** at 0.004 M γ-cyclodextrin; at pH 11: **(m)** at 0.001 M α-cyclodextrin; **(n)** at 0.002 M α-cyclodextrin; **(o)** at 0.004 M α-cyclodextrin; **(p)** at 0.004 M β-cyclodextrin; **(q)** at 0.002 M γ- cyclodextrin. The data were acquired in forward-scattering mode. The insets show the dependence of the main number size distribution peak (nm) on reaction time (min). Samples containing only aggregates were omitted from the main plots but not from the insets.

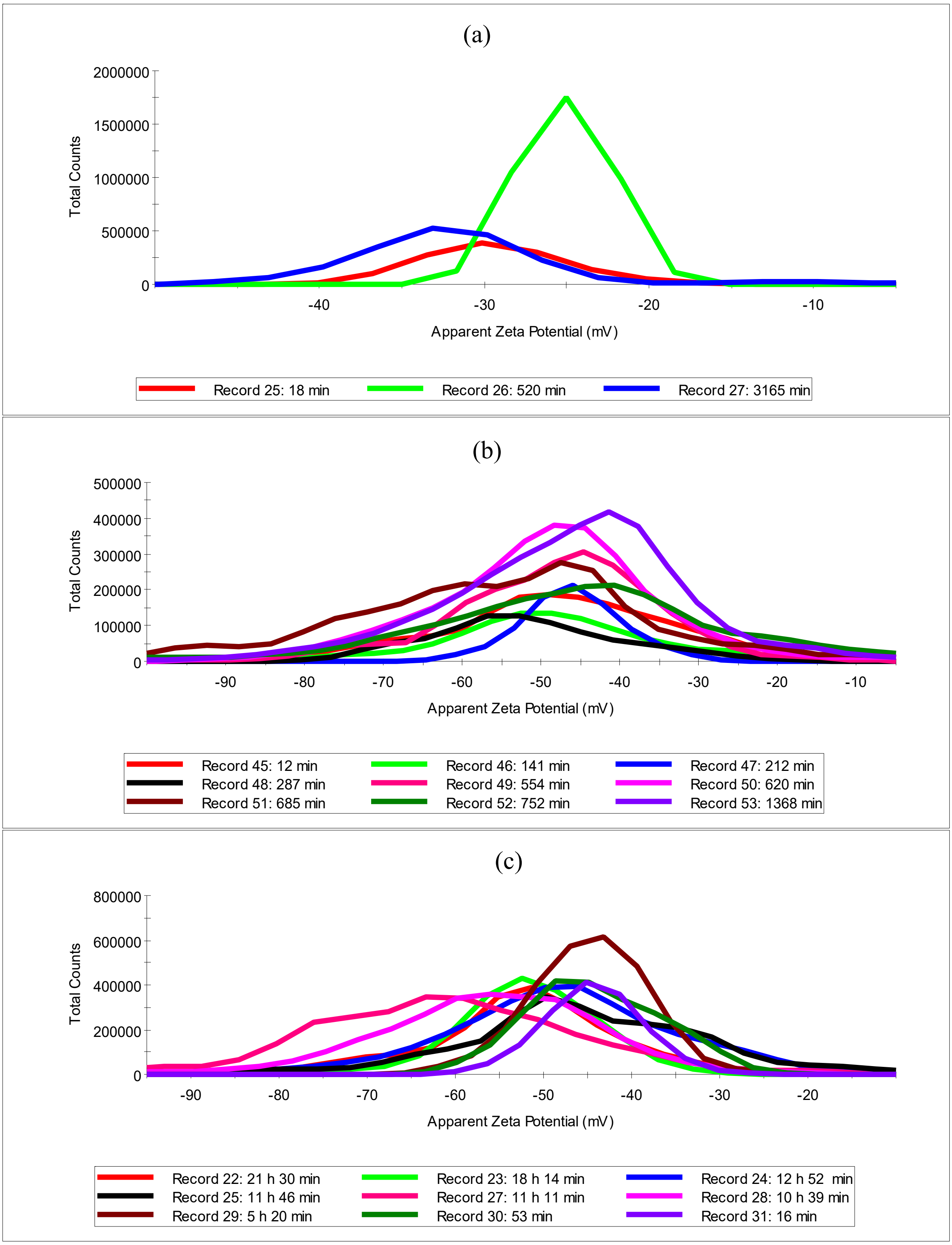

(d)

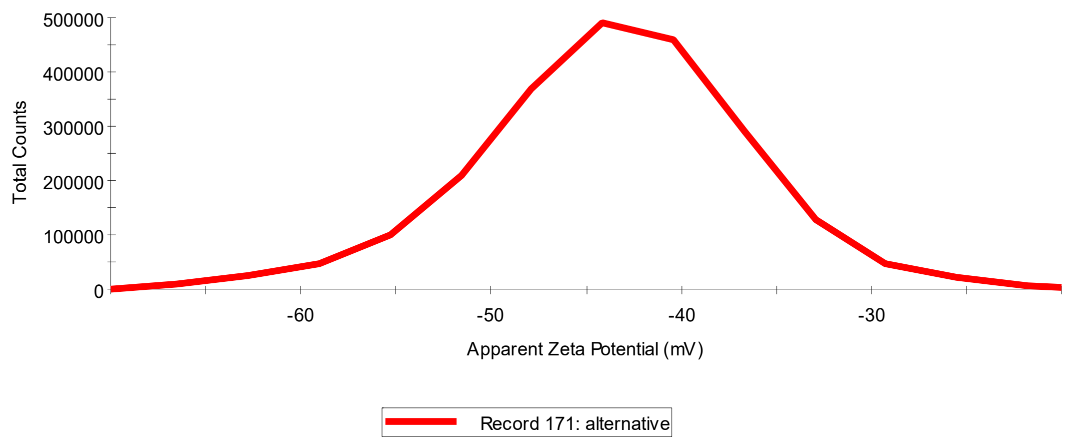
500000
400000
300000
200000
100000
0
Total Counts
-60
-50
-40
-30
Apparent Zeta Potential (mV)
Record 171: alternative

(e)

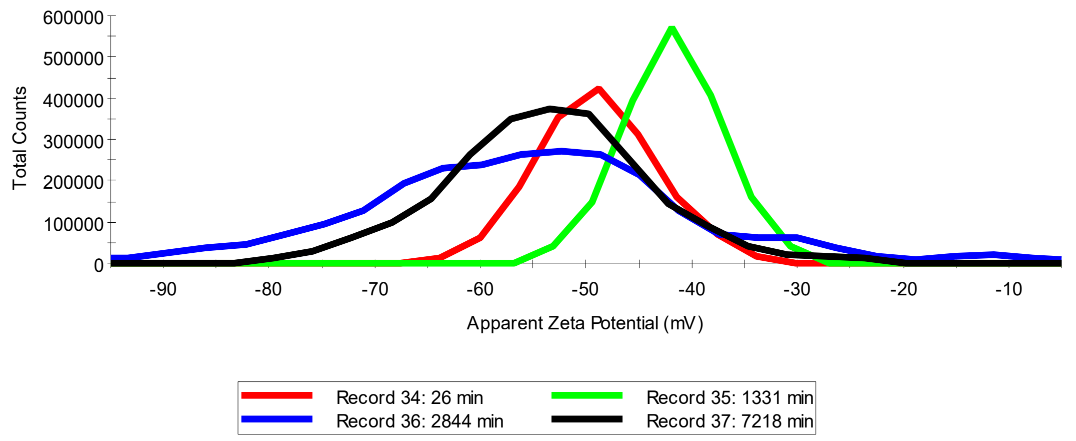
600000
500000
400000
300000
200000
100000
0
Total Counts
-90
-80
-70
-60
-50
-40
-30
-20
-10
Apparent Zeta Potential (mV)
Record 34: 26 min
Record 35: 1331 min
Record 36: 2844 min
Record 37: 7218 min

(f)

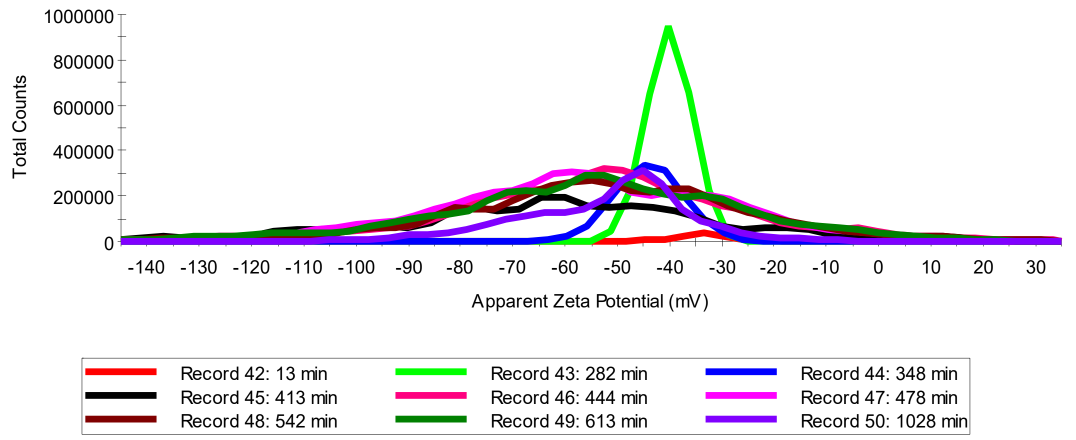
1000000
800000
600000
400000
200000
0
Total Counts
-140 -130 -120 -110 -100 -90 -80 -70 -60 -50 -40 -30 -20 -10 0 10 20 30
Apparent Zeta Potential (mV)
Record 42: 13 min
Record 43: 282 min
Record 44: 348 min
Record 45: 413 min
Record 46: 444 min
Record 47: 478 min
Record 48: 542 min
Record 49: 613 min
Record 50: 1028 min

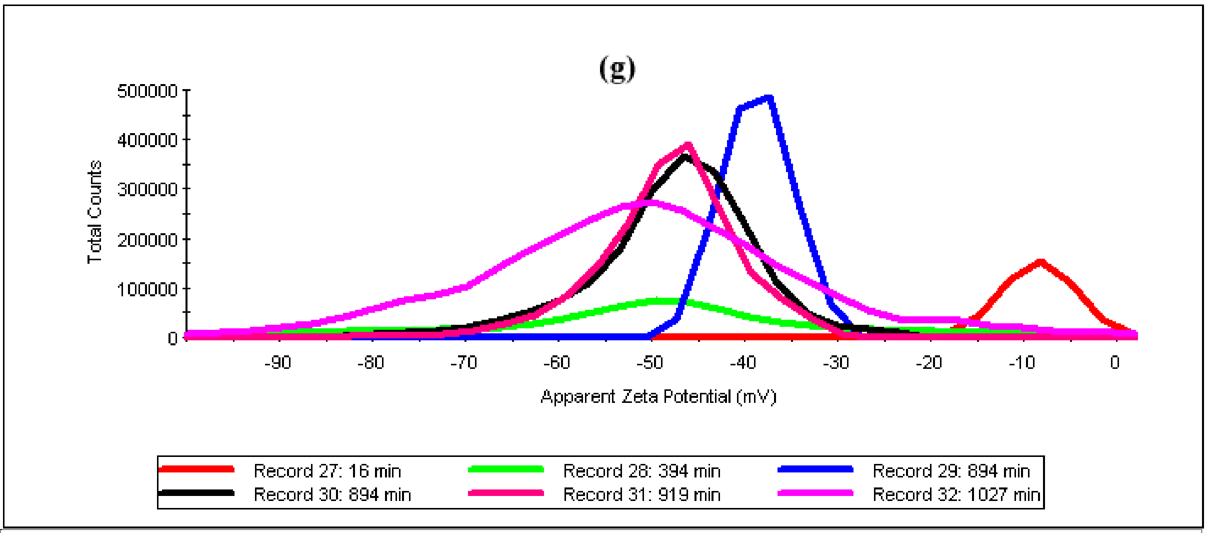
(g)
Total Counts
500000
400000
300000
200000
100000
0
-90 -80 -70 -60 -50 -40 -30 -20 -10 0
Apparent Zeta Potential (mV)
Record 27: 16 min
Record 28: 394 min
Record 29: 894 min
Record 30: 894 min
Record 31: 919 min
Record 32: 1027 min

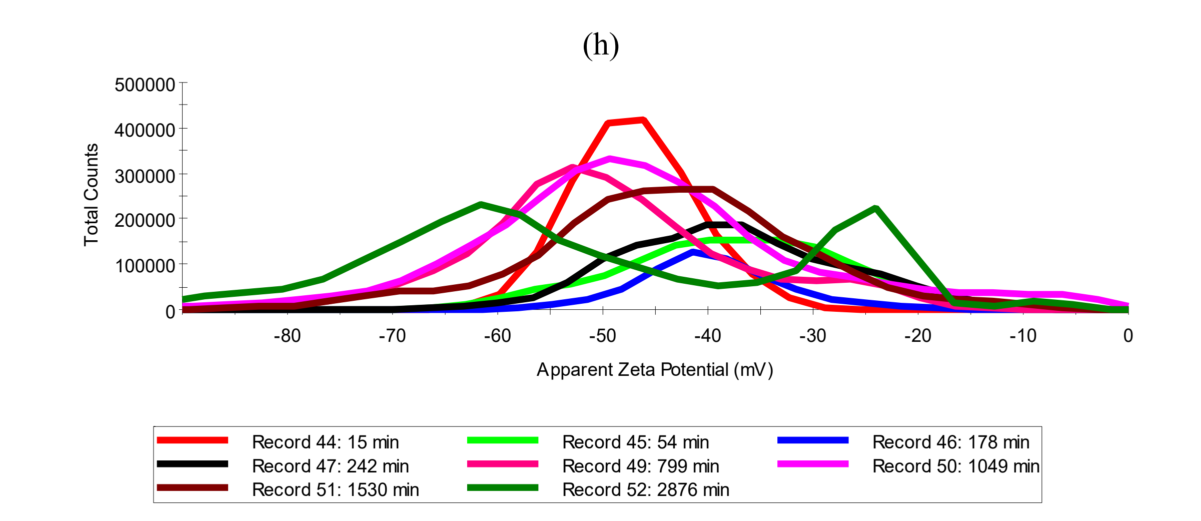
(h)
Total Counts
500000
400000
300000
200000
100000
0
-80 -70 -60 -50 -40 -30 -20 -10 0
Apparent Zeta Potential (mV)
Record 44: 15 min
Record 45: 54 min
Record 46: 178 min
Record 47: 242 min
Record 49: 799 min
Record 50: 1049 min
Record 51: 1530 min
Record 52: 2876 min

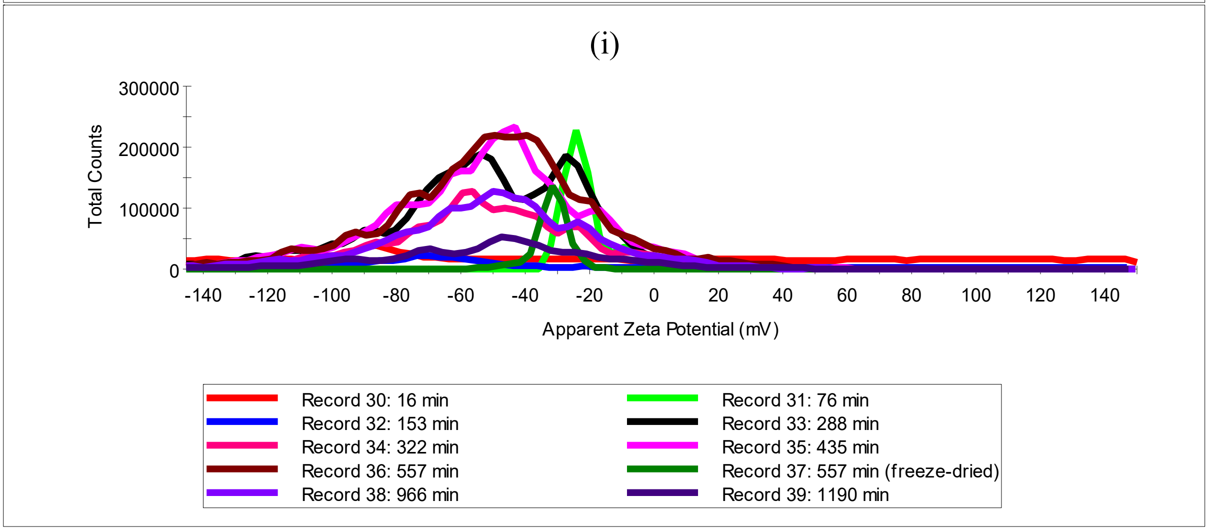
(i)
Total Counts
300000
200000
100000
0
-140 -120 -100 -80 -60 -40 -20 0 20 40 60 80 100 120 140
Apparent Zeta Potential (mV)
Record 30: 16 min
Record 31: 76 min
Record 32: 153 min
Record 33: 288 min
Record 34: 322 min
Record 35: 435 min
Record 36: 557 min
Record 37: 557 min (freeze-dried)
Record 38: 966 min
Record 39: 1190 min

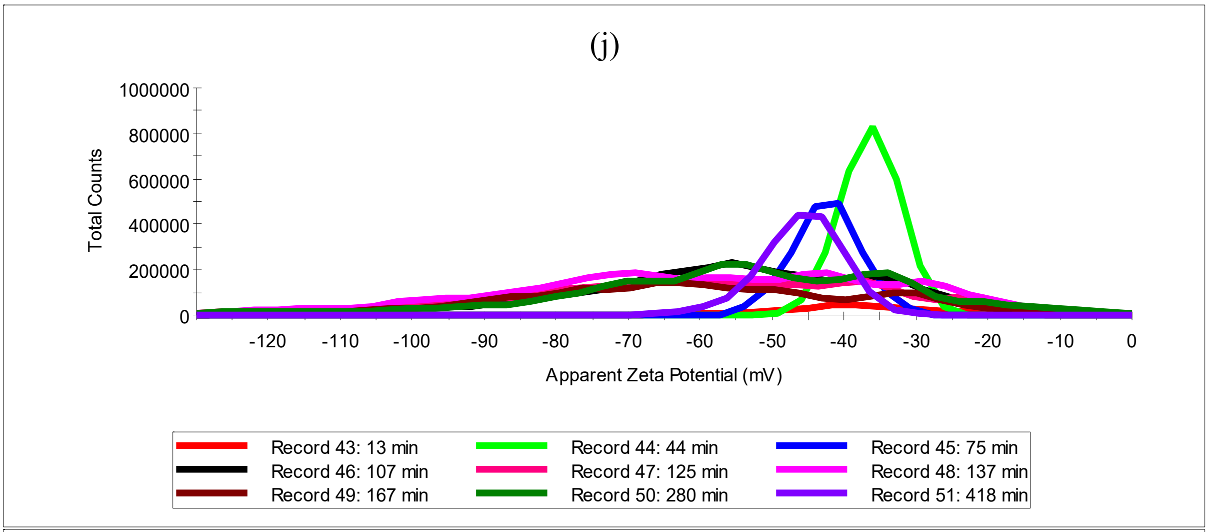
(j)
1000000
800000
600000
400000
200000
0
Total Counts
-120
-110
-100
-90
-80
-70
-60
-50
-40
-30
-20
-10
0
Apparent Zeta Potential (mV)
Record 43: 13 min
Record 44: 44 min
Record 45: 75 min
Record 46: 107 min
Record 47: 125 min
Record 48: 137 min
Record 49: 167 min
Record 50: 280 min
Record 51: 418 min

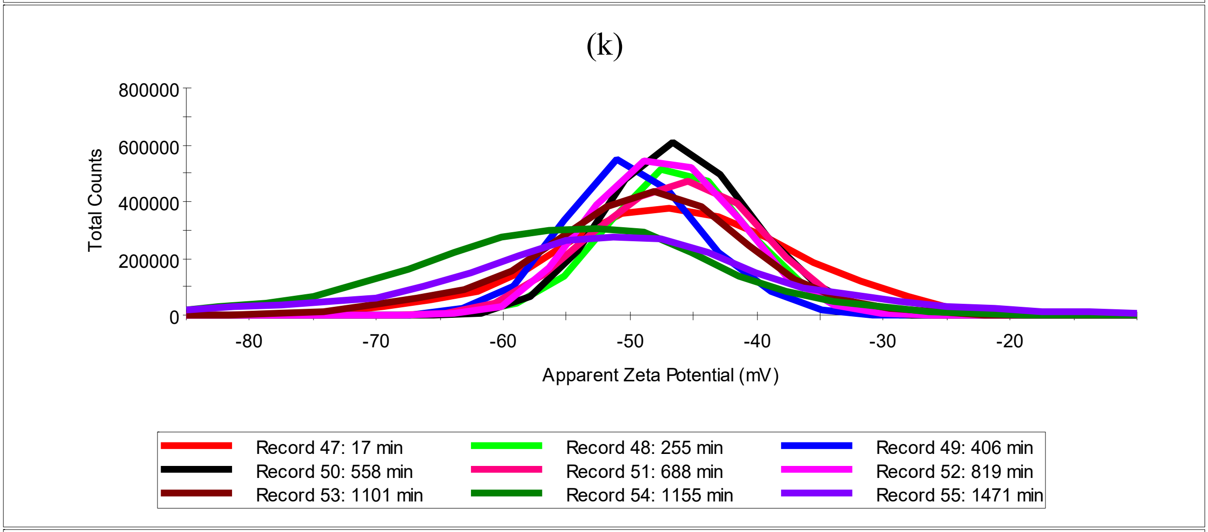
(k)
800000
600000
400000
200000
0
Total Counts
-80
-70
-60
-50
-40
-30
-20
Apparent Zeta Potential (mV)
Record 47: 17 min
Record 48: 255 min
Record 49: 406 min
Record 50: 558 min
Record 51: 688 min
Record 52: 819 min
Record 53: 1101 min
Record 54: 1155 min
Record 55: 1471 min

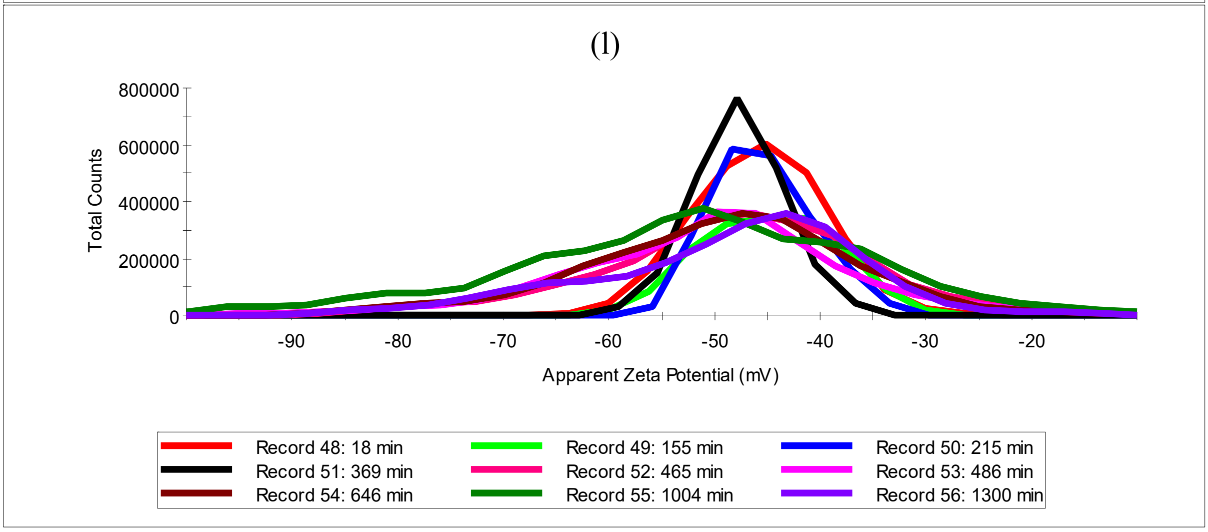
(l)
800000
600000
400000
200000
0
Total Counts
-90
-80
-70
-60
-50
-40
-30
-20
Apparent Zeta Potential (mV)
Record 48: 18 min
Record 49: 155 min
Record 50: 215 min
Record 51: 369 min
Record 52: 465 min
Record 53: 486 min
Record 54: 646 min
Record 55: 1004 min
Record 56: 1300 min

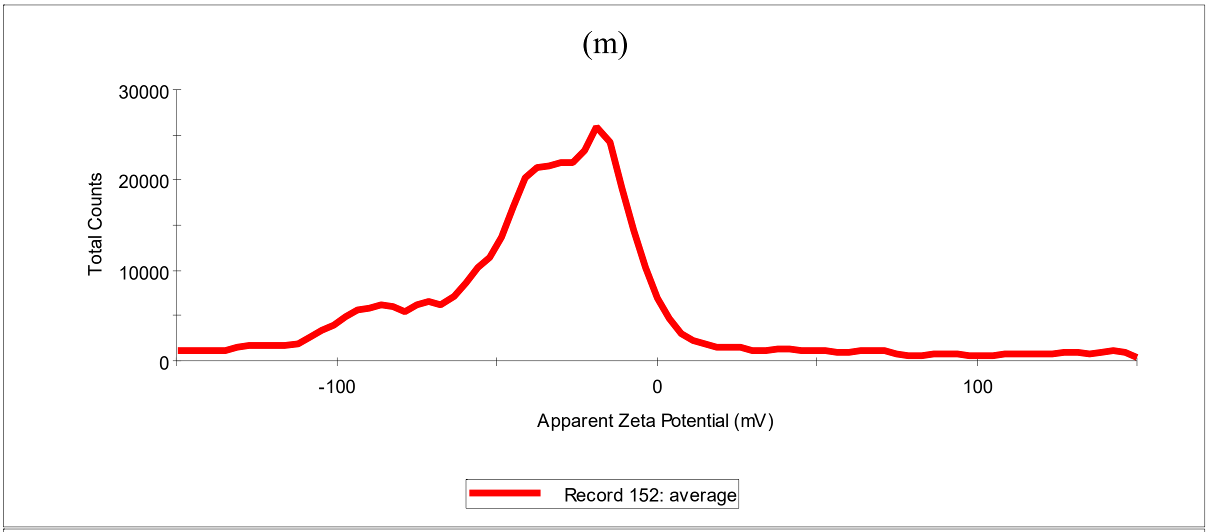
(m)
Total Counts
30000
20000
10000
0
-100
0
100
Apparent Zeta Potential (mV)
Record 152: average

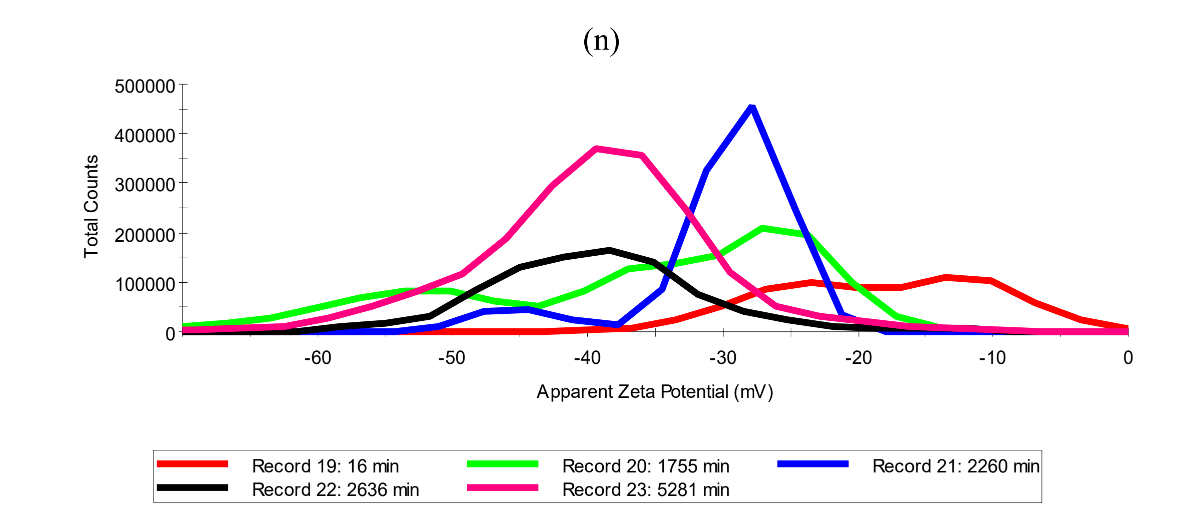
(n)
Total Counts
500000
400000
300000
200000
100000
0
-60
-50
-40
-30
-20
-10
0
Apparent Zeta Potential (mV)
Record 19: 16 min
Record 20: 1755 min
Record 21: 2260 min
Record 22: 2636 min
Record 23: 5281 min

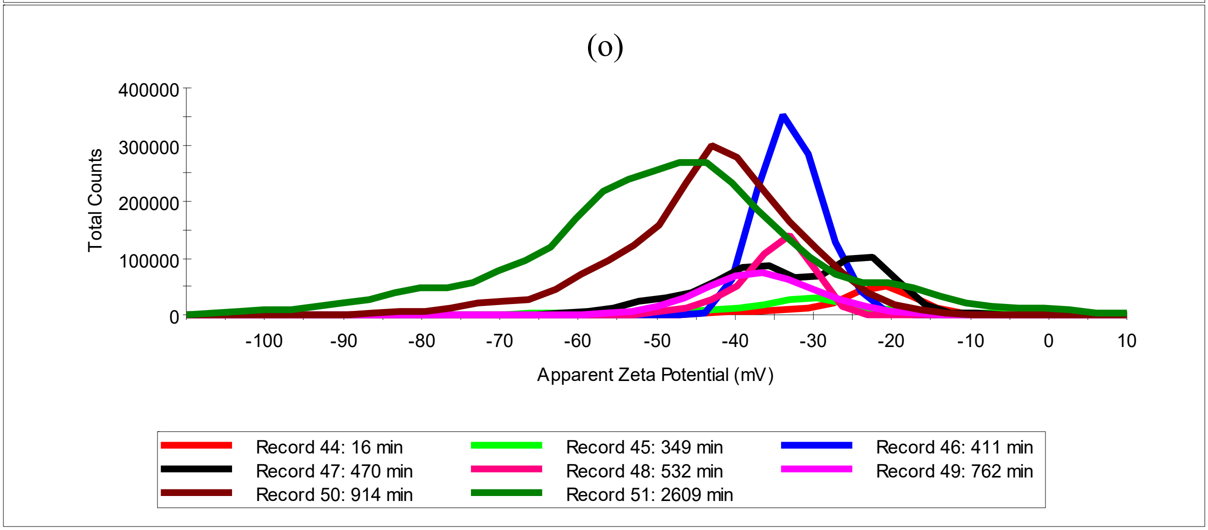
(o)
Total Counts
400000
300000
200000
100000
0
-100
-90
-80
-70
-60
-50
-40
-30
-20
-10
0
10
Apparent Zeta Potential (mV)
Record 44: 16 min
Record 45: 349 min
Record 46: 411 min
Record 47: 470 min
Record 48: 532 min
Record 49: 762 min
Record 50: 914 min
Record 51: 2609 min

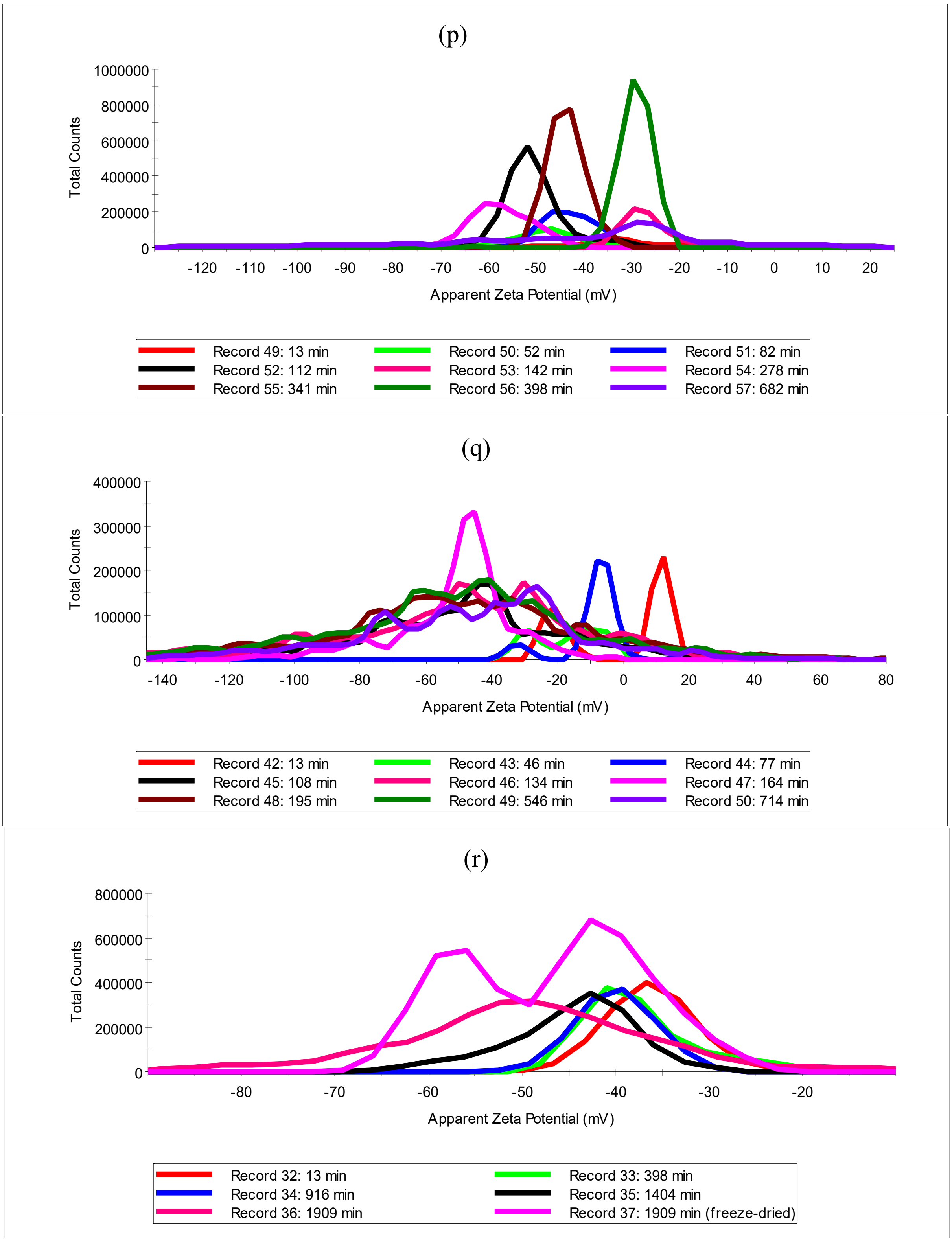

Figure S5. Zeta potential distributions of gold nanoparticles synthesized at 0.1% (w/w) $HAuCl_4$ at room temperature **(a)** at 0.001 M γ-cyclodextrin, pH 10.56; at pH 12: **(b)** at 0.004 M α-cyclodextrin; **(c)** at 0.004 M β-cyclodextrin; **(d)** at 0.004 M γ-cyclodextrin after 18.5 h; at pH 11.5: **(e)** at 0.001 M α-cyclodextrin; **(f)** at 0.002 M α-cyclodextrin; **(g)** at 0.002 M α-cyclodextrin in the absence of HCl; **(h)** at 0.002 M α-cyclodextrin in the presence of NaCl; **(i)** at 0.004 M α-cyclodextrin; **(j)** at 0.004 M β-cyclodextrin; **(k)** at 0.001 M γ-cyclodextrin; **(l)** at 0.002 M γ-cyclodextrin; **(m)** at 0.004 M γ-cyclodextrin after 16 h; at pH 11: **(n)** at 0.001 M α-cyclodextrin; **(o)** at 0.002 M α-cyclodextrin; **(p)** at 0.004 M α-cyclodextrin; **(q)** at 0.004 M β-cyclodextrin; **(r)** at 0.002 M γ-cyclodextrin. Samples containing only aggregates were omitted for clarity.

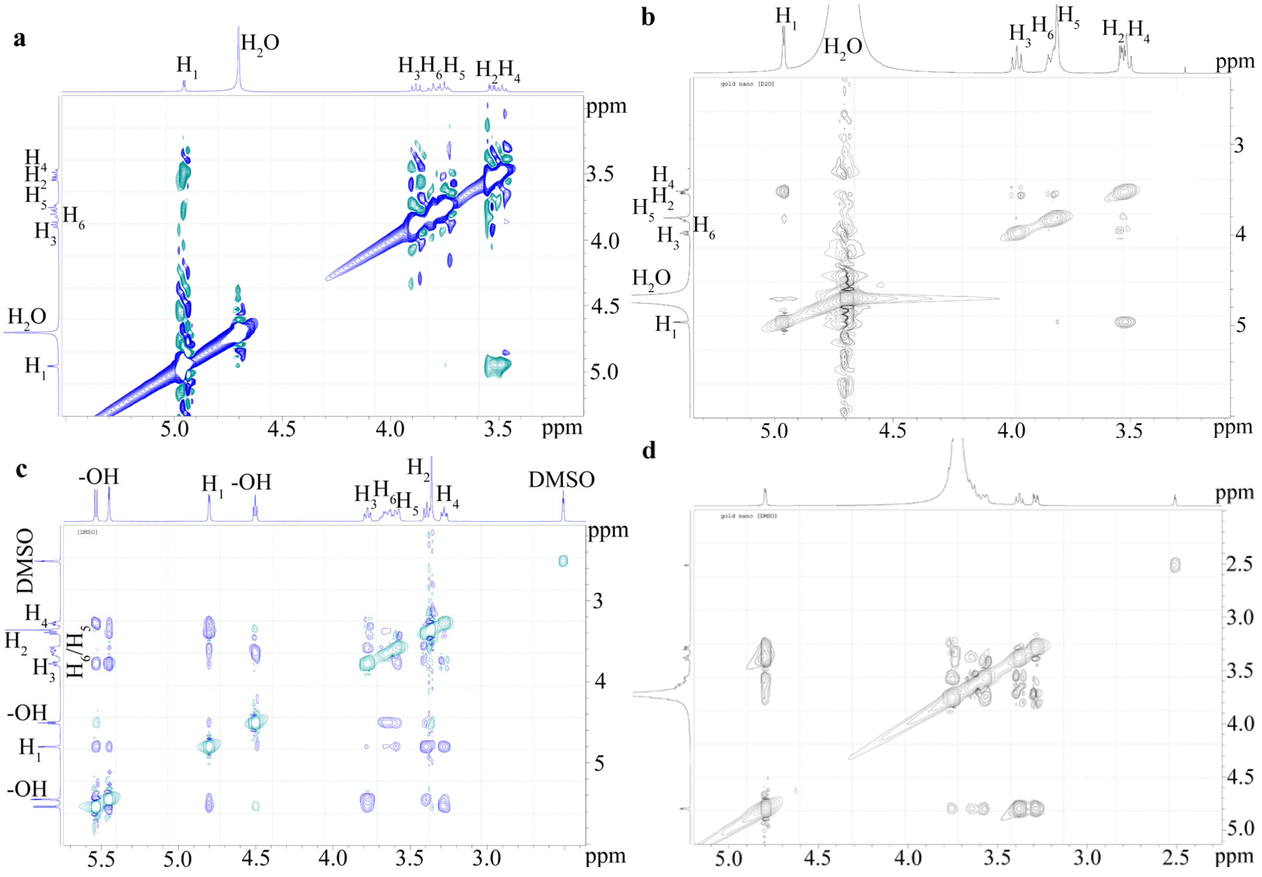

Figure S6. ROESY spectra of α-cyclodextrin alone **(a,c)** and in the presence of $HAuCl_4$ at pH 6.16 **(b,d)** in D_2O **(a,b)** and in DMSO **(c,d).**
